# 

**Published:** 1924-01

**Authors:** 


					CONTENTS. p
i T^e Evolutionary History of Renal Surgery and of Temporal Bone
Surgery
By J. L\cy Firth, M D., M.S. Lond., F.R.C.S.
u?denal Ulcers : Their Detection by Photography.
By T.
Carwardine, M.S.
i6
The Leprosy Problem and its Bearing on Tuberculosis.
By Sir
Leonard Rogers, C.I E., M.D., F.R.C.P., F.R.C.S., F.R.S.,
I-M.S.Ret
19
The Parkinsonian Syndrome : Some Points in Diagnosis and
reatment.
By Macdonald Critchley, M.B., Ch.B.
25
Reviews of Books
34
Editorial Notes
41
Meetings of Societies
42
The L'brary of the Bristol Medico-Chirurgical Society   45
Obituary
Henry Ernest Hetling, L.R.C.P., L.R.C.S. Edin.
47
a' Medical Notes
48

				

